# Caspase-1 affects chronic restraint stress-induced depression-like behaviors by modifying GABAergic dysfunction in the hippocampus

**DOI:** 10.1038/s41398-023-02527-x

**Published:** 2023-06-27

**Authors:** Mingxing Li, Xuejiao Sun, Zongqin Wang, Yi Li

**Affiliations:** 1 Affiliated Wuhan Mental Health Center, Tongji Medical College, Huazhong University of Science and Technology, Wuhan, 430012 China; 2 Department of Psychiatry, Wuhan Mental Health Center, Wuhan, 430012 China; 3 Department of Rehabilitation Medicine, Zhongnan Hospital, Wuhan University, Wuhan, 430071 China

**Keywords:** Depression, Molecular neuroscience

## Abstract

Major depression disorder (MDD) is one of the most common psychiatric disorders and one of the leading causes of disability in worldwide. Both inflammation and GABAergic dysfunction have been implicated in the pathophysiology of MDD. Caspase-1, a classic inflammatory caspase, regulates AMPARs-mediated glutamatergic neurotransmission. However, the role of caspase-1 in chronic stress-induced GABAergic dysfunction remains largely unknown. In this study, we found that serum and hippocampal caspase-1-IL-1β levels increased significantly in chronic restraint stress (CRS) mice, and a significant negative correlation occurred between levels of caspase-1 and depression-like behaviors. Furthermore, CRS significantly decreased GAD67 mRNA levels and GABAergic neurotransmission accompanied by the reduction of GABA concentration, reduced the amplitude and frequency of mIPSCs inhibitory postsynaptic currents (mIPSCs) and the decreased surface expression of GABA_A_Rs γ2 subunit in the hippocampus. Genetic deficiency of caspase-1 not only blocked CRS-induced depression-like behaviors, but also alleviated CRS-induced impairments in GABAergic neurotransmission. Finally, reexpression of caspase-1 in the hippocampus of Caspase-1^−/−^ mice increased susceptibility to stress-induced anxiety- and depression-like behaviors through inhibiting GAD67 expression and GABA_A_Rs-mediated synaptic transmission. Our study suggests that CRS dysregulates GABAergic neurotransmission via increasing the levels of caspase-1-mediated neuroinflammation in the hippocampus, ultimately leading to depression-like behaviors. This work illustrates that targeting caspase-1 may provide potential therapeutic benefits to stress-related GABAergic dysfunction in the pathogenesis of MDD.

## Introduction

Major depression disorder (MDD) is a common and debilitating neurobiological illnesses that affect about 300 million people globally [1]. Although the exact pathophysiological mechanism of MDD is still elusive, emerging evidence elucidates that inflammation has a critical role in the pathophysiology of MDD [2, 3]. Proinflammatory cytokines, such as interleukin (IL)-1β, have shown significant increase in the serum and brain of depressed patients [4, 5]. Caspase-1, also known as IL-1β-converting enzyme, has been linked to the pathological mechanism of MDD [5]. Increased levels of caspase-1 mRNA are observed in periphery blood mononuclear cells (PBMCs) from MDD patients, and can be reduced by treatment with antidepressants [6]. In rodents, chronic stress increases the expression of caspase-1 in brain and peripheral plasma [7, 8]. Furthermore, pharmacological inhibition of caspase-1 prevents chronic restraint stress (CRS)-induced depression-like behaviors via regulating the gut microbiota composition [9]. Our previous study has also demonstrated that genetic deficiency of caspase-1 decreases chronic social defeat stress-induced depression- and anxiety-like behaviors via regulating the stability of surface AMPARs [8]. A recent study reveals that caspase-1 deletion prevents chronic mild stress-induced depression-like behaviors through improving the proptosis of astrocytes in the hippocampus [10]. However, the exact role and precise mechanisms of caspase-1 in CRS-induced depression-like behaviors remains largely unknown.

Accumulating studies have shown that MDD is associated with the GABAergic (gamma-aminobutyric acidergic) dysfunction [11], such as decreased concentration of GABA [12], reduced expression of glutamic acid decarboxylase (GAD) and GABA_A_ receptors (GABA_A_Rs) [13–16]. Interestingly, both selective serotonin reuptake inhibitors (SSRIs) and electroconvulsive therapy produce antidepressant effects via increasing cortical GABA in depressed patients [17, 18]. Furthermore, chronic stress reduces GABAergic neurotransmission in the hippocampus, and antidepressants reverse the GABAergic dysfunction [19]. A recent study reveals that chronic social defeat stress decreases the surface expression of GABA_A_Rs and impairs GABAergic neurotransmission in the ventral hippocampus [20]. GAD synthesizes GABA from glutamate in GABAergic neurons, and two isoforms of GAD65 and GAD67 are separately encoded by the *GAD2* and *GAD1* genes, respectively [21]. Previous studies reveal that the expression of the full-length *GAD1* transcript and GAD67 protein are decreased in the prefrontal cortex and hippocampus of patients with MDD [22–24]. Moreover, chronic stress significantly induces the reductions of GAD67 protein in the prefrontal cortex and hippocampus [15]. However, the cause of GABAergic dysfunction in the pathogenesis of MDD is still obscure.

There is an inextricable regulatory relationship between inflammation and GABAergic neurotransmission in the depression [4]. Previous study has shown that IL-1β inhibits GABA_A_Rs current in cultured hippocampal neurons [25]. In addition, IL-1β has dual effects on GABA_A_Rs-mediated mIPSCs in the central amygdala neurons [26]. IL-1β mRNA in the hippocampus of the post-stroke depression is significantly increased, and the content of GABA in the lateral hypothalamic area is decreased [27]. Based on the above studies, we speculated that caspase-1 may mediate chronic stress-GABAergic dysfunction via regulating the expression of IL-1β pathway in the brain. Moreover, our previous study reveals that suppression of caspase-1-IL-1β provides a defense mechanism against chronic stress-induced glutamatergic dysfunction in the hippocampus [8]. However, the link between caspase-1 and the GABAergic dysfunction in the pathogenesis of MDD remains unknown.

In the present study, we hypothesized that CRS upregulated the levels of caspase-1, subsequently impaired GABA_A_Rs neurotransmission via reducing the GAD67 expression, eventually resulting in depression-like behaviors. Therefore, we use a variety of behavioral tests, stereotactic injection, electrophysiological recordings, immunofluorescence, and molecular experiments to determine the effects of caspase-1 on CRS-induced depression-like behaviors and GABAergic dysfunction. Our data provide the clarification of the possible molecular mechanism for GABAergic dysfunction in the pathogenesis of MDD.

## Materials and methods

### Animals

Adult male C57BL/6 J mice (8–10 weeks) were purchased from Beijing Vitalriver Laboratory Animal Corp. Ltd (Beijing, China). Caspase-1^−/−^ and their wild-type (WT) mice on a C57BL/6 J background were originally purchased from Jackson Laboratories (Bar Harbor, Maine, USA). *Caspase-1*^−/−^ mice and their wild-type (WT) littermates (8–10 weeks aged mice) were used in this study. For *Caspase-1*^+/−^ mice genotyping, reverse transcriptase-polymerase chain reaction (RT-PCR) were performed with 2 × Taq PCR Reaction Mix (KT201, TIANGEN Biotech, Beijing, China) with the following primers: 5′-GAAGAGATGTTACAGAAGCC-3′ (forward), 5′-CATGCCTGAATAATGATCACC-3′ (reverse). The mice were housed in standard laboratory conditions (22 ± 2 °C; 12 h light/dark cycle) with free access to food and water unless otherwise indicated. All animal procedures were conducted in accordance with the National Institutes of Health Guide for the Care and Use of Laboratory Animals and approved by the Animal Welfare Committee of Huazhong University of Science and Technology.

### Drugs

Tetrodotoxin (TTX), AP-5, CNQX, sodium L-ascorbate and dimethyl sulfoxide (DMSO) were purchased from Sigma-Aldrich (Saint Louis, USA). Other general agents were purchased from commercial suppliers.

### Stress procedures

CRS was performed as our previously reported [7, 28]. Mice were subjected to restraint stress for 2 h/day in 50 ml conical tubes with ventilation holes for 21 days. After restraint stress, the mice were immediately returned to their home cages. Control mice remained in their home cages.

Subthreshold variable stress (STVS) was used to assess stress susceptibility according to previous studies [29]. Mice were subjected to 100 random mild foot shocks at 0.45 mA for 1 h, a tail suspension stress for 1 h and restraint stress in a 50 ml conical tubes for 1 h. This protocol was processed for 3 days.

### Sucrose preference test (SPT)

SPT was used to assess anhedonia according to previously established protocols [8, 30]. The mice were subjected to two bottles filled with either 1% sucrose or water following 24 h period of food and water deprivation. The bottle position was randomly switched to prevent the position preference effect in the mice, and the weight of bottles was recorded and changed every 12 h during the test. Sucrose preference (%) was equal to sucrose consumption/ total consumption × 100%.

### Tail suspension test (TST)

TST was used to measure despair/depression-like behaviors as previously described [8, 31, 32]. Mice were individually suspended from a strap and hung for 6 min from the tip of the tail fixed 20 cm above the floor. Immobility was considered a lack of all movements, except for the need to move and breathe. Observers were blind to the treatment of mice.

### Forced swim test (FST)

FST was performed to measure depression-like behaviors as previously described [8, 28, 32]. Mice were individually put into a glass cylinder (35 × 15 cm diameter) containing 15 cm water (22 ± 1 °C). The mice were allowed to swim for 6 min, and the final 4 min interval of the test was recorded. Immobility was considered the minimal movements to keep its head above the water or floating. Observers were blind to the treatment of mice.

### Elevated-plus maze (EPM)

EPM was used to measure anxiety-like behaviors as previously described [8, 33]. EPM consisted of two closed arms and two open (30 × 5 × 0.5 cm), which were up to 40 cm above the floor. The mice were placed in the center are and allowed to explore for 5 min. The AniLab video motility system (AniLab, Ningbo, China) was used to evaluate the anxiety-like behavior of mice by calculating the time spent in the open arm and the total number of entries into the open arm.

### Open filed (OF)

C57BL/6 J mice were placed individually into the activity chamber (50 × 50 × 40 cm) and monitored by using AniLab video motility system (AniLab, Ningbo, China). Total distance and average speed in the OF test were recorded and assessed for 10 min in the activity chamber.

### Enzyme-linked immunosorbent assay (ELISA)

Abdominal aortic blood samples were collected and centrifuged at 3000 *g* for 10 min at 4 °C, and the supernatant was collected and stored at −80 °C until use. The concentration of corticosterone (E-OSEL-M0001, Elabscience, Wuhan, China), caspase-1 (E-EL-M0201c, Elabscience, Wuhan, China), and IL-1β (E-EL-M0037c, Elabscience, Wuhan, China) was quantified by using a commercially available ELISA kit according to the manufacturer’s instructions. Hippocampus were weighted, homogenized and centrifuged at 8000 g for 10 min, and then GABA levels in hippocampal supernatants were detected according to the ELISA kit instructions (E-BC-K852-M, Elabscience, Wuhan, China).

### Quantitative real-time PCR (qPCR)

Total RNA was isolated and quantified as previously described [8]. The purified total RNAs (1000 ng) were reverse-transcribed to cDNA using the RevertAid First Strand cDNA Synthesis Kit (K1622, ThermoFisher Scientific, USA) according to the manufacturer’s instructions. qPCR was performed on LightCycler® 96 Instrument System (Roche, Mannheim, Germany) with FastStart Essential DNA Green Master (Roche, Mannheim, Germany). The Amplification conditions were as follows: 95 °C for 10 min followed by 40 cycles of 95 °C for 15 s, 60 °C for 30 s and 72 °C for 30 s. Relative target gene mRNA expression was normalized to glyceraldehyde-3-phosphate dehydrogenase (GAPDH) mRNA and calculated using the ΔΔCt method. Primer sequences were listed in Supplementary Table 1.

### Western blotting

Western blotting was performed according to the previous studies [8, 34]. The hippocampal slices were homogenized in RIPA buffer and protein concentrations were quantified with coomassie blue protein-binding assay. The proteins (30 μg) in each sample were separated by 10% SDS-PAGE. After blocking with 5% BSA for 1 h at room temperature, the membranes were incubated overnight at 4 °C with the primary antibodies: β-actin (1:3000, abs137975, absin, China); GAD67 (1:500, MAB5406, Merk Millipore, USA); caspase-1 (1:1000, 06-503-I, Merk Millipore, USA); GABA_A_Rs γ2 (1:400, bs-4112R, Bioss, China); GAPDH (1:3000, abs132004, absin, China); parvalbumin (PV) (1:200; bs-1299R, Bioss, China). The secondary HRP-conjugated antibodies were incubated for 1 h at room temperature with shaking. The membranes were visualized using an enhanced chemiluminescence (ECL) (Super Signal West Pico; Pierce Chemical Co., Rockford, USA) and images were captured by G:BOX Chemi XRQ (Syngene, Jerusalem, UK). NIH ImageJ software was used to analyzed the relative quantity of the bands and β-actin or GAPDH was used as loading control. All assays were performed at least three times.

### Biotinylation of surface proteins

Surface protein extraction was performed according to the previous studies [20]. Hippocampal slices were incubated with aCSF containing 1.0 mg/ml sulfo-NHS-LC-biotin (21335, Thermo Scientific, Rockford, USA) for 1 h and then terminated with 7.5 mg/ml glycine for 20 min, and subsequently lysed in the buffer. The supernatant was quantified and equal amounts of protein were incubated with Agarose Resin (29201, Thermo Scientific, Rockford, USA) overnight at 4 °C. Biotinylated surface or total proteins were measured with western blotting.

### Whole-cell patch-clamp recordings

Whole-cell patch-clamp recordings was performed as our previously reported [8, 20]. Mice were anaesthetized with sodium pentobarbital (45 mg/kg, intraperitoneally (i.p.)) and perfused with cutting-solution (in mM): 209 sucrose, 3.1 sodium pyruvate, 22 glucose, 1.25 NaH_2_PO_4_, 12 sodium L-ascorbate, 4.9 MgSO_4_•7H_2_O and 26 NaHCO_3_; oxygenated with 95% O_2_ and 5% CO_2_, pH 7.2-7.4. The coronal hippocampal slices (300 μm) were cut by a vibratome (VT1000S, Leica, Germany), and the slices were incubated for at least 1.5 h in an interface chamber containing aCSF. Whole-cell voltage clamp recordings of CA1 pyramidal cells were made in a submersion chamber. For GABA_A_Rs-mediated mIPSCs recordings, patch pipettes (3–6 MΩ) were filled with solution containing (in mM): 140 CsCl, 0.1 CaCl_2_, 2 MgCl_2_, 10 HEPES, 10 EGTA, 4 K_2_ATP, pH 7.2 (280–300 mOsm).

GABA_A_Rs-mediated mIPSCs were recorded in the presence of CNQX (10 μM), AP5 (50 μM) and TTX (1 μM). All recordings were performed at a holding potential of −70 mV with a Multiclamp 700B amplifier and Digidata 1322 A digitizer (Molecular Devices, Sunnyvale, CA, USA) at room temperature, and then was acquired with pClamp 10 software. Data was analyzed by Mini Analysis Program (Synaptosoft, Decatur, GA, USA).

### Plasmid construction and stereotaxic delivery

Recombinant adenovirus-associated virus (rAAV) vectors encoding the genes for caspase-1 (NCBI CCDS ID CCDS22798.1) or GFP were purchased from BrainVTA (BrainVTA Co., Ltd., Wuhan, China). Mice were anesthetized with sodium pentobarbital (45 mg/kg, i.p.) and infused bilaterally with 1.5 μl of rAAV virus (3.4 × 10^12^ genome copies/ml) into the hippocampus CA1 region (AP = − 2.0 mm, ML = ± 1.2 mm, DV = − 2.0 mm) using a stereotaxic injector (Ruiwode Life Science, Shenzhen, China). Behavioral testing and biochemical assays were performed at 3 weeks after viral injection. Fluorescence microscopy and western blotting were performed to estimate the effects of transfection in vivo.

### Immunofluorescence and immunohistochemistry

Immunohistochemistry was performed according to our previous studies [8, 35]. The mice were anesthetized with sodium pentobarbital (45 mg/kg, i.p.) and perfused intracardially with 0.9% saline followed by 4% paraformaldehyde. Hippocampal slices (30 μm) were obtained from a freezing microtome (CM1900, Leica Microsystems, Wetzlar, Germany). For immunofluorescence, slices were loaded on slides and imaged using a Nikon microscopy (Digital Cameral DXM 1200, Nikon, Tokyo, Japan).

For immunohistochemistry, slices were incubated in room temperature with 3% hydrogen peroxide (H_2_O_2_) for 30 min and blocked with 1% BSA. The slices were incubated overnight at 4 °C with PV (1:50; bs-1299R, Bioss, China), and subsequently exposed to biotinylated goat anti-rabbit IgG using an avidin-biotin complex kit. Finally, PV-positive GABAergic interneurons were visualized with 3, 3´-diaminobenzidine (Sigma-Aldrich, USA). Slices were stained with hematoxylin and imaged using a PANNORAMIC MIDI (3D HISTECH, Hungary). No immunoreactivity was observed in the negative controls.

### Statistical analysis

All data were presented as the mean ± SEM. Statistical analyses were performed using GraphPad Prism software. Statistical differences were evaluated using the Student’s *t*-tests, two-way analysis of variance (ANOVA) with Bonferroni’s multiple comparison test, or repeated-measures ANOVA. Statistical significance was set at *p* < 0.05.

## Results

### Chronic stress upregulates the expression of caspase-1 and impairs GABAergic neurotransmission in the hippocampus

**Fig. 1:**
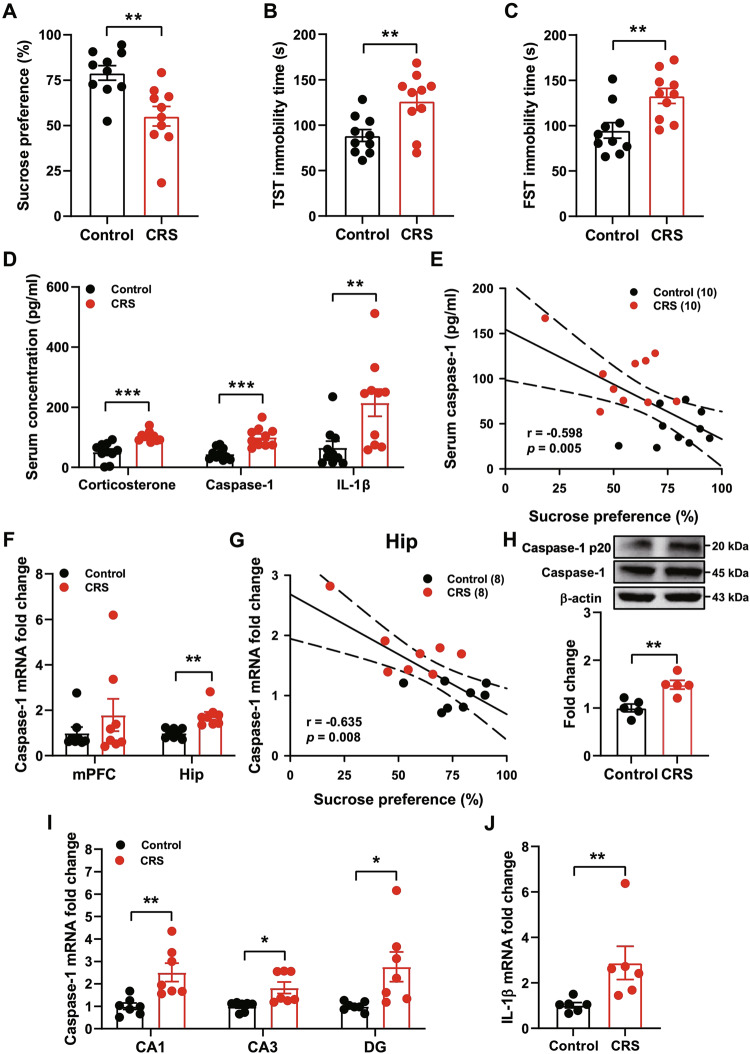
CRS upregulates the expression of caspase-1. **A–C** CRS induced anhedonia in SPT, and increased immobility time in TST and FST (*n* = 10 mice/group, Student’s test). **D** CRS increased the levels of corticosterone, caspase-1 and IL-1β in the serum (*n* = 10 mice/group, Student’s test). **E** Correlation of serum caspase-1 levels with sucrose preference after CRS (Pearson correlation). **F** Caspase-1 mRNA in the medial prefrontal cortex (mPFC) and hippocampus (Hip) were determined by qPCR after CRS (*n* = 8 mice/group, Student’s test). **G** Correlation of caspase-1 levels in the Hip with sucrose preference after CRS (Pearson correlation). **H** Representative immunoblots and quantification of caspase-1 levels in the Hip from Control and CRS mice (*n* = 5 mice/group, Student’s test). **I** Caspase-1 mRNA in the CA1, CA3 and DG of hippocampus (*n* = 7 mice/group, Student’s test). **J** CRS increased the levels of IL-1β mRNA in the hippocampus (*n* = 6 mice/group, Student’s test). All data are shown as means ± SEM. ^*^p < 0.05, ^**^p < 0.01 and ^***^p < 0.001.

Compared with control mice, CRS mice exhibited decreased sucrose preference in SPT (Fig. 1A) and increased immobility time in TST (Fig. 1B) and FST (Fig. 1C). These results demonstrated that CRS induced significant depression-like behaviors. Next, we found that CRS significantly increased the serum levels of corticosterone, caspase-1 and IL-1β (Fig. 1D). Furthermore, there was significant negative correlation between the caspase-1 and sucrose preference (Fig. 1E). In order to validate this finding, we further analyzed the expression of caspase-1 in the medial prefrontal cortex (mPFC) and hippocampus (Hip) by qPCR, and found that CRS selectively upregulated the caspase-1 expression in the hippocampus, but not mPFC (Fig. 1F). Furthermore, there was also significant negative correlation between the caspase-1 and sucrose preference in the hippocampus (Fig. 1G). Furthermore, CRS mice showed a significant increase in the levels of the activated form of caspase-1 protein in the hippocampus compared with control mice (Fig. 1H). Meanwhile, the increased levels of caspase-1 displayed no more subfield specificity among CA1, CA3 and DG of the hippocampus (Fig. 1I). Interestingly, CRS mice also showed a significant increase in the IL-1β expression in the hippocampus (Fig. 1J). These results suggest that chronic stress may upregulate caspase-1-mediated neuroinflammation in the hippocampus.

GABAergic dysfunction is known to contribute to the pathophysiology of depression [11], and inflammation may severely impair GABAergic pathways, resulting in depression-like behaviors [4]. Therefore, we analyzed the two isoforms of GAD65 and GAD67, as well as vesicular GABA transporter (VGAT) mRNA levels, which were involved in GABA synthesis and transport.

**Fig. 2:**
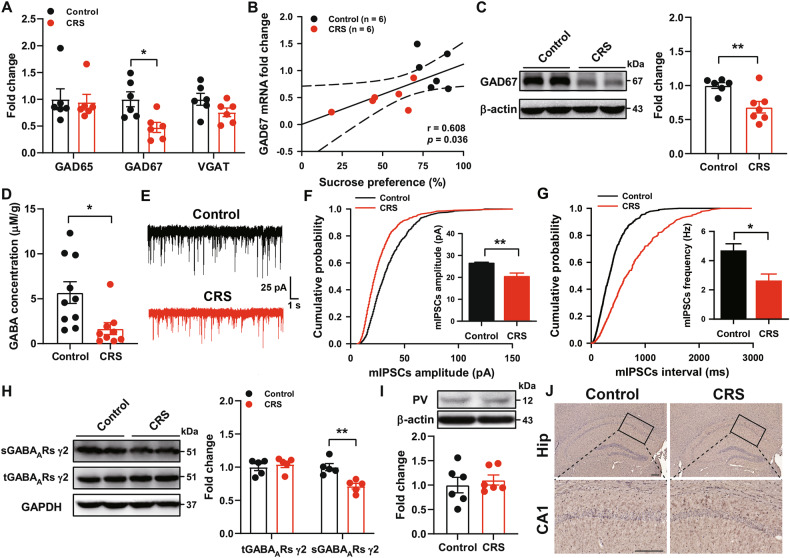
CRS impairs GABAergic neurotransmission in the hippocampus. **A** Hippocampal GAD65, GAD67 and VGAT mRNA were determined by qPCR after CRS (*n* = 6 mice/group, Student’s test). **B** Correlation of the levels of GAD67 mRNA with sucrose preference after CRS (Pearson correlation). **C** Representative immunoblots and quantification of GAD67 levels from Control and CRS mice (*n* = 6-7 mice/group, Student’s test). **D** CRS reduced the levels of GABA in the hippocampus (*n* = 9-10 mice/group, Student’s test). **E** Representative whole-cell voltage-clamp traces of GABA_A_Rs-mediated mIPSCs in the pyramidal neurons of hippocampus from Control and CRS mice. Scale bars: 25 pA, 1 s. **F**, **G** Amplitude and frequency of GABA_A_Rs-mediated mIPSCs from Control and CRS mice (*n* = 8-9 cells from 3-4 mice/group, Student’s test). **H** Representative immunoblots and quantification of the surface levels of GABA_A_Rs γ2 subunit (sGABA_A_Rs γ2) and total levels of GABA_A_Rs γ2 subunit (tGABA_A_Rs γ2) in the hippocampus from Control and CRS mice (*n* = 5 mice/group, Student’s test). **I** Representative immunoblots and quantification of parvalbumin (PV) levels from Control and CRS mice (*n* = 5 mice/group, Student’s test). **J** Immunohistochemistry against PV-positive GABAergic interneurons in the hippocampus. Scale bars: 200 μm. All data are shown as means ± SEM. ^*^p < 0.05 and ^**^p < 0.01.

CRS selectively reduced GAD67 mRNA levels, but not GAD65 and VGAT (Fig. 2A). Further, GAD67 gene expression strongly correlated with sucrose preference (Fig. 2B). Similarly, CRS also significantly downregulated hippocampal GAD67 protein expression (Fig. 2C). Meanwhile, CRS decreased the levels of GABA in the hippocampus (Fig. 2D). Next, we measured GABA_A_Rs-mediated mIPSCs in CA1 pyramidal neurons. The results showed that both the amplitude and frequency of mIPSCs decreased significantly in CRS mice compared to control mice (Fig. 2E–G), and there were no effects of CRS on the mIPSCs rise time and decay time (Supplementary Fig. 1). In addition, CRS significantly reduced the surface levels of GABA_A_Rs γ2 subunit, with no effect on the total levels of GABA_A_Rs γ2 subunit (Fig. 2H), which was consistent with the electrophysiological results. However, there was no significant difference in the expression of PV and the number of PV-positive GABAergic interneurons in the hippocampus (Fig. 2I–J). These indicate that CRS inhibits the GABAergic neurotransmission via reducing the expression of GAD67.

Taken together, these results suggest that chronic stress upregulates the expression of caspase-1-IL-1β pathway in the serum and hippocampus, and further impairs hippocampal GABAergic neurotransmission via promoting caspase-1-mediated neuroinflammation.

### Caspase-1 deletion blocks CRS-induced depression-like behaviors

**Fig. 3:**
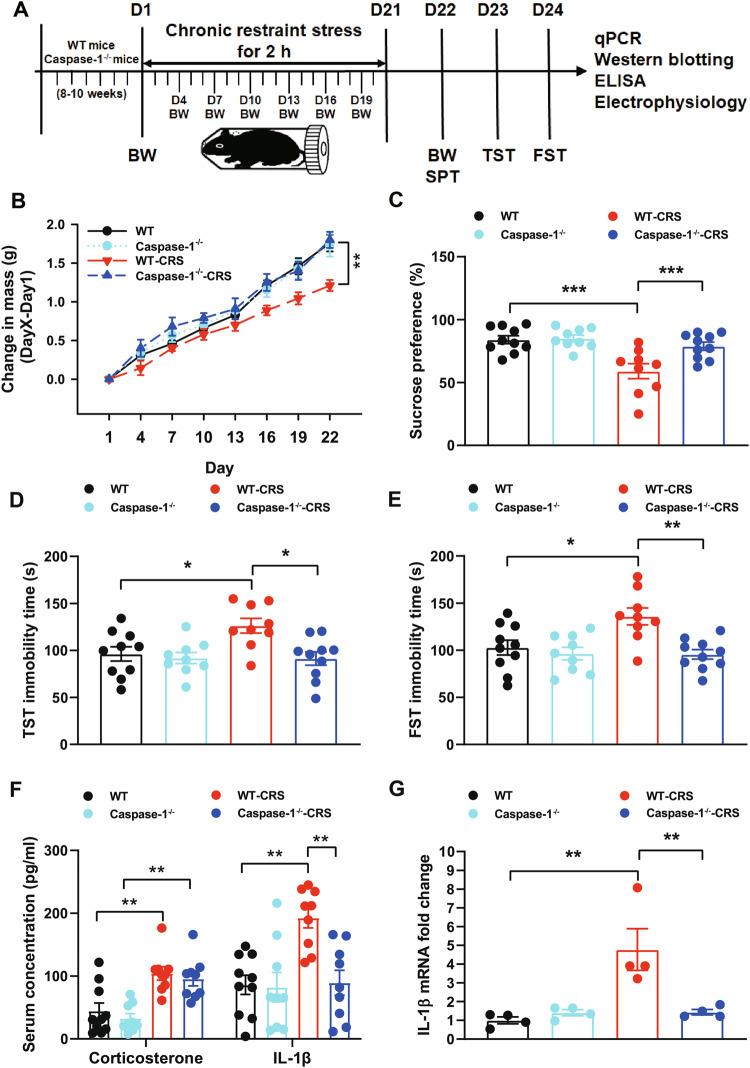
Caspase-1 deletion blocks CRS-induced depression-like behaviors. **A** A schematic diagram depicting the experimental procedure. D, day. **B** Body weight throughout the CRS treatment (*n* = 9-10 mice/group, repeated-measures ANOVA: CRS, F_(7,238)_ = 2.301, *p* < 0.05; Genotype, F_(7,238)_ = 1.345, *p* > 0.05; Interaction, F_(7,238)_ = 2.101, *p* < 0.05). **C–E** Gene ablation of caspase-1 prevented CRS-induced anhedonia in SPT (two-way ANOVA: CRS, F_(1,34)_ = 15.934, *p* < 0.001; Genotype, F_(1,34)_ = 7.217, *p* < 0.05; Interaction, F_(1,34)_ = 5.783, *p* < 0.05), and increased immobility time in TST (two-way ANOVA: CRS, F_(1,34)_ = 4.154, *p* < 0.05; Genotype, F_(1,34)_ = 7.372, *p* < 0.05; Interaction, F_(1,34)_ = 4.441, *p* < 0.05) and FST (two-way ANOVA: CRS, F_(1,34)_ = 4.995, *p* < 0.05; Genotype, F_(1,34)_ = 10.452, *p* < 0.001; Interaction, F_(1,34)_ = 5.490, *p* < 0.05) (*n* = 9-10 mice/group, two-way ANOVA, Bonferroni’s test). **F** Serum corticosterone (CRS, F_(1,33)_ = 32.745, *p* < 0.001; Genotype, F_(1,33)_ = 0.904, *p* > 0.05; Interaction, F_(1,33)_ = 0.02, *p* > 0.05) and IL-1β (CRS, F_(1,33)_ = 9.160, *p* < 0.01; Genotype, F_(1,33)_ = 8.120, *p* < 0.01; Interaction, F_(1,33)_ = 7.058, *p* < 0.05) from WT, WT-CRS, Caspase-1^−/−^ and Caspase-1^−/−^-CRS groups (*n* = 9-10 mice/grou*p*, two-way ANOVA). **G** Caspase-1 deletion blocked CRS-induced increase of IL-1β mRNA in the hippocam*p*us (*n* = 4 mice/group, two-way ANOVA: CRS, F_(1,12)_ = 61.957, *p* < 0.001; Genotype, F_(1,12)_ = 37.686, *p* < 0.001; Interaction, F_(1,12)_ = 58.990, *p* < 0.001). All data are shown as means ± SEM. ^*^p < 0.05, ^**^p < 0.01, and ^***^p < 0.001.

To determine the effect of caspase-1 on GABAergic dysfunction induced by CRS, caspase-1 knockout mice were used (Fig. 3A and Supplementary Fig. 2A). Compared with non-stressed control mice, WT-CRS mice showed reduced body weight gain, and this reduction was completely prevented in the *Caspase-1*^−/−^ mice (Fig. 3B). Different from WT-CRS mice, *Caspase-1*^−/−^ mice exposed to CRS showed increased sucrose consumption (Fig. 3C) and decreased immobility time in TST (Fig. 3D) and FST (Fig. 3E), which were consistent with our previous results [8]. Furthermore, *Caspase-1*^−/−^ mice blocked CRS-induced elevation of IL-1β in the serum, but not corticosterone (Fig. 3F). Meanwhile, CRS upregulated the levels of IL-1β mRNA in the hippocampus of WT mice, but not *Caspase-1*^−/−^ mice (Fig. 3G). These results suggest that caspase-1 deletion blocks CRS-induced depression-like behaviors through inhibiting neuroinflammation in the hippocampus.

### Caspase-1 deficiency prevents CRS-induced reduction of GABA_A_Rs-mediated synaptic transmission

**Fig. 4:**
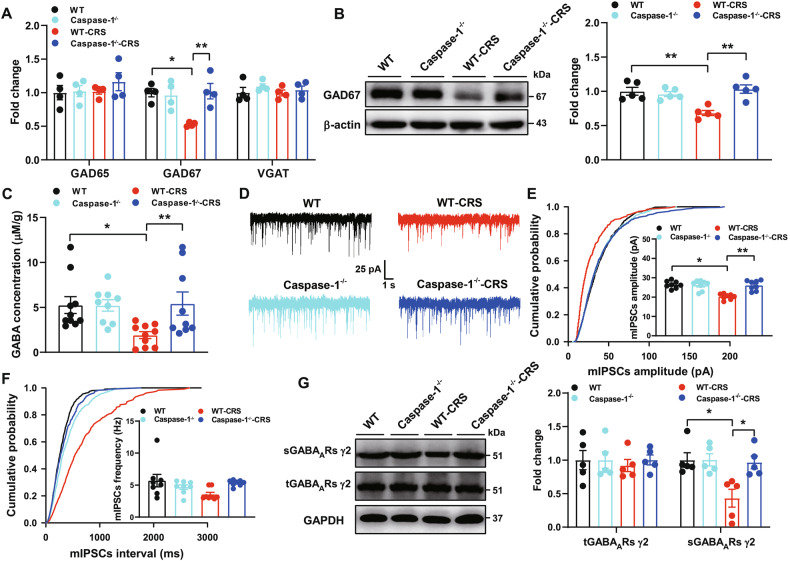
Caspase-1 deficiency prevents CRS-induced reduction of GABA_A_Rs-mediated synaptic transmission in the hippocampus. **A** Hippocampal GAD65 (two-way ANOVA: CRS, F_(1,12)_ = 0.671, *p* > 0.05; Genotype, F_(1,12)_ = 0.735, *p* > 0.05; Interaction, F_(1,12)_ = 0.392, *p* > 0.05), GAD67 (two-way ANOVA: CRS, F_(1,12)_ = 6.604, *p* < 0.05; Genotype, F_(1,12)_ = 7.439, *p* < 0.05; Interaction, F_(1,12)_ = 10.025, *p* < 0.01) and VGAT (two-way ANOVA: CRS, F_(1,12)_ = 0.277, *p* > 0.05; Genotype, Genotype, F_(1,12)_ = 1.501, *p* > 0.05; Interaction, F_(1,12)_ = 0.193, *p* > 0.05) mRNA were determined by qPCR from WT, WT-CRS, Caspase-1^−/−^ and Caspase-1^−/−^-CRS groups (*n* = 4 mice/group, two-way ANOVA, Bonferroni’s test). **B** Representative immunoblots and quantification of GAD67 levels from different groups (*n* = 5 mice/group, two-way ANOVA: CRS, F_(1,16)_ = 6.024, *p* < 0.05; Genotype, F_(1,16)_ = 9.735, *p* < 0.01; Interaction, F_(1,16)_ = 15.582, *p* < 0.01). **C** Caspase-1 deficiency prevented CRS-induced reduction of GABA in the hippocampus (*n* = 9-10 mice/group, two-way ANOVA: CRS, F_(1,34)_ = 5.861, *p* < 0.05; Genotype, F_(1,34)_ = 6.634, *p* < 0.05; Interaction, F_(1,34)_ = 6.466, *p* < 0.05). **D** Representative whole-cell voltage-clamp traces of GABA_A_Rs-mediated mIPSCs in the pyramidal neurons of hippocampus from different groups. Scale bars: 25 pA, 1 s. **E**, **F** Amplitude (two-way ANOVA: CRS, F_(1,28)_ = 16.962, *p* < 0.001; Genotype, F_(1,28)_ = 13.275, *p* < 0.01; Interaction, F_(1,28)_ = 15.973, *p* < 0.001) and frequency (two-way ANOVA: CRS, F_(1,28)_ = 1.887, *p* > 0.05; Genotype, F_(1,28)_ = 0.482, *p* > 0.05; Interaction, F_(1,28)_ = 6.481, *p* < 0.05) of GABA_A_Rs-mediated mIPSCs from different groups (n = 8 cells from 3-4 mice/group, two-way ANOVA, Bonferroni’s test). **G** Representative immunoblots and quantification of sGABA_A_Rs γ2 (CRS, F_(1,16)_ = 7.821, *p* < 0.05; Genotype, F_(1,16)_ = 6.178, *p* < 0.05; Interaction, F_(1,16)_ = 5.994, *p* < 0.05) and tGABA_A_Rs γ2 (CRS, F_(1,16)_ = 0.124, *p* > 0.05; Genotype, F_(1,16)_ = 0.140, *p* > 0.05; Interaction, F_(1,16)_ = 0.143, *p* > 0.05) levels in the hip*p*ocampus from different groups (*n* = 5 mice/group, two-way ANOVA). All data are shown as means ± SEM. ^*^p < 0.05 and ^**^p < 0.01.

To investigate the role of caspsase-1 in GABAergic neurotransmission, we first examined the levels of GAD65, GAD67 and VGAT mRNA in the hippocampus. The GAD67 mRNA levels were markedly reduced in the WT-CRS group but remained unaffected in the *Caspase-1*^−/−^-CRS group (Fig. 4A). No significant differences were observed in GAD65 and VGAT mRNA levels (Fig. 4A). We then measured the protein levels of GAD67 in the hippocampus. *Caspase-1*^−/−^ mice completely blocked the reduction of GAD67 caused by CRS in WT mice (Fig. 4B), indicating caspase-1 deletion may prevent CRS-induced GABAergic dysfunction. As shown by ELISA, caspase-1 deficiency prevented CRS-induced reduction of GABA in the hippocampus (Fig. 4C). Furthermore, WT mice exposed to CRS showed a greatly decreased mIPSCs amplitude and frequency (Fig. 4D–F), which remained unchanged in the *Caspase-1*^−/−^-CRS mice. No effects were found in the mIPSCs rise time and decay time (Supplementary Fig. 2B–C). Moreover, CRS significantly reduced the surface levels of GABA_A_Rs γ2 subunit in WT but not in *Caspase-1*^−/−^ mice (Fig. 4G). Taken together, the above results showed that deficiency of caspase-1 may prevent CRS-induced GABAergic dysfunction via restoring the expression of GAD67 in the hippocampus.

### Overexpression of caspase-1 in the hippocampus increases susceptibility to stress-induced anxiety- and depression-like behaviors

**Fig. 5:**
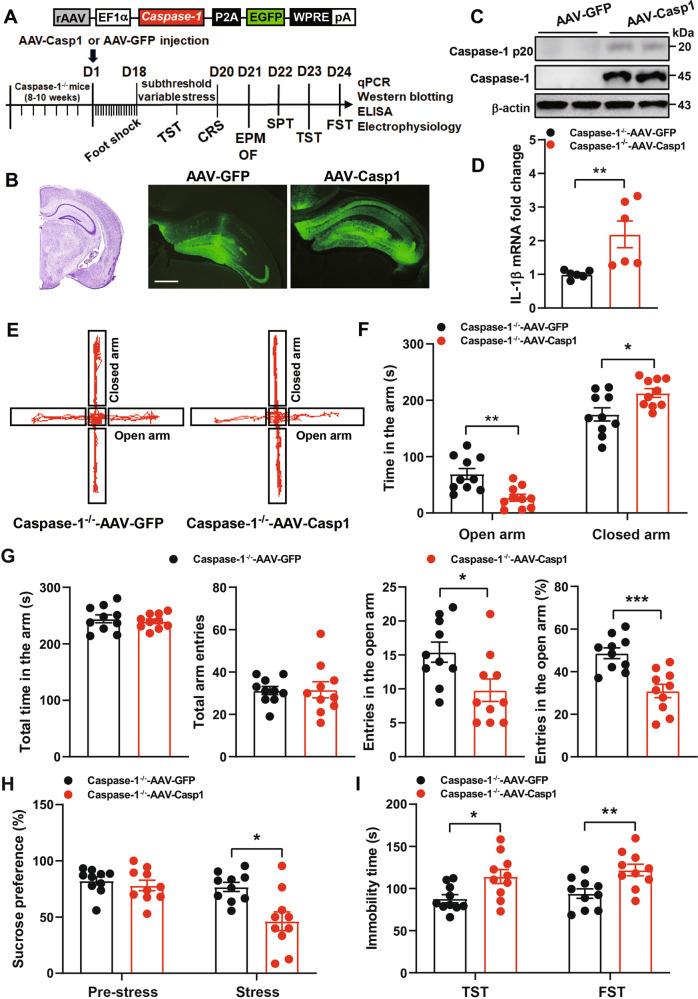
Overexpression of caspase-1 in the hippocampus increases susceptibility to stress-induced anxiety- and depression-like behaviors. **A** Top, schematic representation of AAV-based constructs for overexpression of caspase-1. Bottom, the timeline of experimental procedure. **B** Representative fluorescence of injection sites in the hippocampus of Caspase-1^−/−^ mice. Scale bars: 200 μm. **C** Representative immunoblots of hippocampal caspase-1 after subthreshold variable stress (STVS). **D** AAV-Casp1-injected mice showed increased IL-1β mRNA in the hippocampus after STVS (*n* = 6 mice/group, Student’s test). **E–G** AAV-Casp1-injected mice displayed an anxiogenic phenotype in EPM after STVS (*n* = 10 mice/group, Student’s test). **H** AAV-Casp1-injected mice decreased sucrose preference after STVS (*n* = 10 mice/group, repeated-measures ANOVA, Bonferroni’s test). **I** AAV-Casp1-injected mice showed an increase in time spent immobile in the TST and FST after STVS (*n* = 10 mice/group, Student’s test). All data are shown as means ± SEM. ^*^p < 0.05, and ^**^p < 0.01.

To confirm the role of caspase-1 in the CRS-induced GABAergic dysfunction, we tested if enhanced caspase-1 expression in the hippocampus of Caspase-1^−/−^ mice could induce depression-like behaviors and GABAergic dysfunction. We used adeno-associated virus to reexpression caspase-1 in the hippocampus (Fig. 5A). The accuracy of the injection site was validated with immunofluorescence staining of enhanced green fluorescent protein (GFP), and a significant upregulation of caspase-1 and IL-1β in the hippocampus was observed (Fig. 5B–D). AAV-Casp1 mice displayed an anxiogenic phenotype following subthreshold variable stress (STVS), which was not sufficient to induce depression- or anxiety-associated behavior [29]. Compared with AAV-GFP mice, AAV-Casp1 mice spent less time in the open arms and conversely more time in the closed arms (Fig. 5E, F), and fewer visits and ratio of visits in the open arms (Fig. 5E, G). In addition, there was no significant differences in the OF test (Supplementary Fig. 3). Further, AAV-Casp1 mice displayed decreased sucrose preference (Fig. 5H) and increased immobility time in the TST and FST (Fig. 5I) compared with AAV-GFP mice. These results clearly demonstrate that reexpression of caspase-1 in the hippocampus of Caspase-1^−/−^ mice is sufficient to induce anxiety- and depression-like behaviors.

### Reexpression of caspase-1 in the hippocampus reduces the GABA_A_Rs-mediated synaptic transmission

**Fig. 6:**
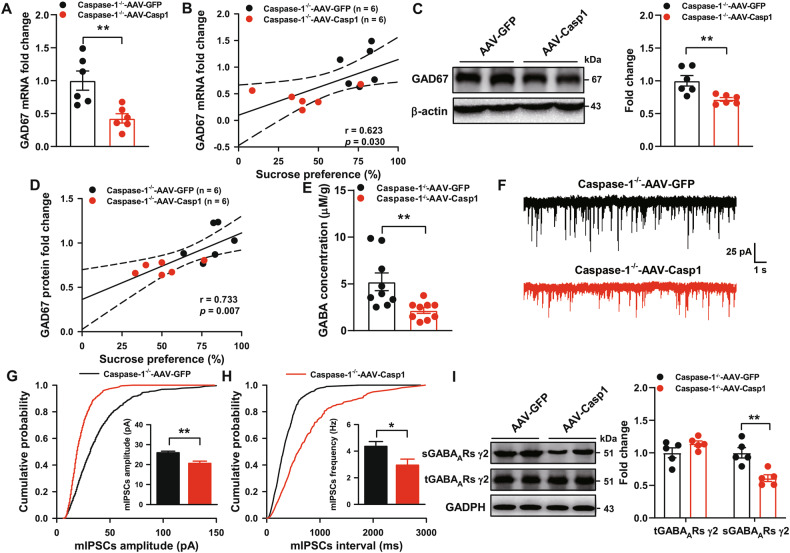
Reexpression of caspase-1 in the hippocampus reduces the GABA_A_Rs-mediated synaptic transmission. **A** Hippocampal GAD67 was determined by qPCR from AAV-GFP and AAV-Casp1-injected mice after STVS (*n* = 6 mice/group, Student’s test). **B** Correlation of hippocampal GAD67 mRNA levels with sucrose preference after STVS (Pearson correlation). **C** Representative immunoblots and quantification of GAD67 protein expression in the hippocampus (*n* = 6 mice/group, Student’s test). **D** Correlation of hippocampal GAD67 protein expression with sucrose preference after STVS (Pearson correlation). **E** GABA levels in the hippocampus after STVS (*n* = 9 mice/group, Student’s test). **F** Representative whole-cell voltage-clamp traces of GABA_A_Rs-mediated mIPSCs in the pyramidal neurons of hippocampus from AAV-GFP and AAV-Casp1-injected mice after STVS. Scale bars: 25 pA, 1 s. **G**, **H** Amplitude and frequency of GABA_A_Rs-mediated mIPSCs (*n* = 8 cells from 3-4 mice/group, Student’s test). **I** Representative immunoblots and quantification of sGABA_A_Rs γ2 and tGABA_A_Rs γ2 levels in the hippocampus (*n* = 5 mice/group, Student’s test). All data are shown as means ± SEM. ^*^p < 0.05 and ^**^p < 0.01.

We next evaluated whether overexpression of caspase-1 could alter GABAergic neurotransmission. AAV-Casp1 infusion resulted in significantly decreased levels of GAD67 mRNA after STVS (Fig. 6A). Further, GAD67 gene expression strongly correlated with sucrose preference (Fig. 6B). Similarly, AAV-Casp1 mice also showed a significant decrease in GAD67 protein expression (Fig. 6C). In addition, there was significant correlation between the GAD67 protein expression and sucrose preference (Fig. 6D). Interestingly, AAV-Casp1 mice showed a significant reduction of GABA in the hippocampus (Fig. 6E). Meanwhile, the whole-cell patch clamp experiment showed that overexpression of caspase-1 in the hippocampus significantly decreased the mIPSCs amplitude and frequency (Fig. 6F–H), with no effect on mIPSCs rise time and decay time (Supplementary Fig. 4). Furthermore, STVS significantly reduced the surface levels of GABA_A_Rs γ2 subunit in AAV-Casp1 mice (Fig. 6I). Taken together, these results further suggest that overexpression of caspase-1 in the hippocampus of Caspase-1^−/−^ mice increases susceptibility to stress through impairing GABAergic neurotransmission.

## Discussion

In this study, we demonstrated that the levels of caspase-1-IL-1β pathway were increased in CRS mice and negatively correlated with depression-like behaviors. Furthermore, caspase-1-mediated neuroinflammation impaired GABAergic neurotransmission via reducing the expression of GAD67 in the hippocampus. Gene deficiency of caspase-1 in mice rescued CRS-induced decrease in surface levels of GABA_A_Rs γ2 subunit and GABA_A_Rs-mediated mIPSCs by inhibiting the production of IL-1β, and subsequently produced antidepressant-like effects. Moreover, reexpression of caspase-1 in the hippocampus of Caspase-1^−/−^ mice reversed these effects. To the best of our knowledge, our results provided a direct relationship between caspase-1-mediated neuroinflammation and chronic stress-induced GABAergic dysfunction (Supplementary Fig. 5), and further identifying caspase-1 as a novel target for treatment of MDD.

Both clinical and preclinical findings have consistently demonstrated that hippocampus and prefrontal cortex are vulnerable to chronic stress [36, 37]. Furthermore, the ventral hippocampus input to medial prefrontal cortex (mPFC) has been implicated in the pathophysiological mechanisms of MDD [38]. Thus, we measured the changes in expression of caspase-1 in these brain regions of mice. Surprisingly, the levels of caspase-1 were strikingly elevated in the hippocampus, but there was no significant difference in the mPFC. This is consistent with preclinical studies that chronic mild stress selectively increases the protein levels of caspase-1 in the hippocampus [39]. Moreover, our previous study found chronic social defeat stress selectively increases the levels of caspase-1 in the hippocampus [8]. These results reveal specific alterations in caspase-1 expression at the hippocampus in depressed mice and suggest that differences in its expression may have a key role in the pathophysiology of MDD.

Increased circulatory cytokine levels have been observed both in MDD patients and depressive rodent models [4, 5]. It is well known that caspase-1 is the most typical inflammatory mediator, and regulates the production of IL-1β and IL-18. Interestingly, IL-1β and IL-18 are significantly increased in patients with MDD, and their levels are associated with the severity of depression [6]. In agreement with the key role of caspase-1 in the MDD [5, 8], in our study, CRS significantly increased the expression of caspase-1-IL-1β in the serum and hippocampus, and genetic deficiency of caspase-1 prevented CRS-induced depression-like behaviors via inhibiting the expression of IL-1β. Reexpression of caspase-1 in the hippocampus of Caspase-1^−/−^ mice was sufficient to induce anxiety- and depression-like behaviors. Although CRS significantly increase the levels of corticosterone [40], we found that knockout of caspase-1 could not prevent CRS-induced increase in serum levels of corticosterone. There is no direct evidence that caspase-1 regulates GABAergic neurotransmission, however, IL-1β has an important role in the regulation of chronic stress-induced glutamatergic and GABAergic neurotransmission in the hippocampus [8, 41]. Here, we found that CRS impaired GABAergic neurotransmission through upregulating caspase-1-IL-1β signaling pathway in the hippocampus. To our knowledge, this is the first study reveal that caspase-1 induces chronic stress-induced depression-like behaviors via impairing the GABAergic neurotransmission in the hippocampus.

Numerous reports of GABAergic abnormalities in the MDD brain [37]. These dysfunctions are potentially the consequence of decreased levels of GAD [42], which is the rate-limiting enzyme for GABA synthesis from glutamate. In this study, our results showed that CRS selectively decreased GAD67 mRNA and protein levels in the hippocampus, without an effect on the GAD65. This is not only consistent with postmortem studies showing reductions in GAD67 mRNA levels in the hippocampus of MDD patients [23], but also with findings in animal models of depression [15, 16]. Moreover, GAD65 synthesizes GABA for vesicular release in an activity-dependent manner, whereas GAD67 maintains basal GABA levels [43]. Consistent with this functional distinction, GAD65 deficient mice show few behavioral abnormality and no change in GABA levels in the brain [44]. However, loss of GAD67 in neurons induces learning and social behavior deficits in mice [45]. A recent study reveals that global knockout of GAD67 elicits emotional abnormality in mice [46]. Furthermore, decreased GABA levels are observed both in patients with MDD and depressive rodent models [12, 47]. Consistent with these findings, our results further support the hypothesis that chronic stress decreases GAD67 expression, and subsequently resulted in the reduction of total GABA content and GABAergic dysfunction.

Our results further revealed that caspase-1 deletion could prevent the decrease in GABA_A_Rs-mediated synaptic transmission in the hippocampus induced by CRS. Interestingly, IL-1β inhibits GAD expression at the mRNA and protein levels in rat islets [48]. Our previous study reveals that chronic stress induces the expression of IL-1β by activating caspase-1 in the hippocampus [8]. In present study, the increased caspase-1 in the hippocampus leads to a decrease in GAD67 via upregulating the expression of IL-1β pathway, thus impairing GABA_A_Rs-mediated mIPSCs and reducing the GABA concentration and surface expression of GABA_A_Rs γ2 subunit. Furthermore, homozygous deletion of *Gad1* significantly reduces the amplitude and frequency of mIPSCs and GABA levels in cultured hippocampal neurons [49]. Moreover, the γ2 subunit is essential for GABA_A_Rs synaptic localization, and the surface expression of GABA_A_Rs γ2 subunit in the hippocampus is decreased by chronic stress [20]. Consistent with these findings, our results further support the existence of an imbalance between glutamatergic and GABAergic neurotransmission in the hippocampus of MDD.

Although we found a correlation between caspase-1 and GABAergic dysfunction, there was no evidence that caspase-1 can directly regulate GABAergic neurotransmission, and the mechanism by which caspase-1 modulates the GABAergic dysfunction needs to be further investigated. Furthermore, we found a reduction of the surface levels of GABA_A_Rs γ2 subunit induced by CRS, however, other subunit combinations that are involved in the decreased of the amplitude and frequency of mIPSCs also needs to be investigated. Interestingly, caspase-1 are present in the neurons, microglia, and astrocyte in the hippocampus [10, 50, 51]. Further studies also need to investigate the role of hippocampal caspase-1 in chronic stress-induced depression by using conditional caspase-1 knockout mice.

In summary, our results provide new evidence for further understanding the inflammation and dysregulation of GABAergic neurotransmission in the pathophysiological mechanism of MDD, and highlights caspase-1 as a potential novel therapeutic target for the treatment of MDD.

## Supplementary information

Supplementary Information

## Data Availability

The data used in this study are available from the corresponding author upon reasonable request.
