# Supplementary material for: Caspase-1 affects chronic restraint stress-induced depression-like behaviors by modifying GABAergic dysfunction in the hippocampus
Source: Transl Psychiatry. 2023 Jun 27;13:229. doi: 10.1038/s41398-023-02527-x (PMC10300106; doi:10.1038/s41398-023-02527-x)
Supplement: Supplementary file 1 — Supplementary Information [file 41398_2023_2527_MOESM1_ESM.doc]

**Supplementary Information for**

**Caspase-1 affects chronic restraint stress-induced depression-like behaviors by modifying GABAergic dysfunction in the hippocampus**

Mingxing Li^1, 2, 3, *^, Xuejiao Sun^3^, ^4^, Zongqin Wang^1, 2^, Yi Li^1, 2, *^

^1^Affiliated Wuhan Mental Health Center, Tongji Medical College, Huazhong University of Science and Technology, Wuhan 430012, China.

^2^Department of Psychiatry, Wuhan Mental Health Center, Wuhan 430012, China.

^3^These authors contributed equally: Mingxing Li, Xuejiao Sun.

^4^Department of Rehabilitation Medicine, Zhongnan Hospital, Wuhan University, Wuhan 430071, China.

*Correspondence: Dr. Mingxing Li and Prof. Yi Li, Affiliated Wuhan Mental Health Center, Tongji Medical College of Huazhong University of Science and Technology, No. 89 Gonglongbing Road, Wuhan 430012, China.

E-mail addresses: [china123li@aliyun.com](mailto:china123li@aliyun.com) (Mingxing Li), (psylee@163.com, Yi Li).

**Supplementary Figures**

**
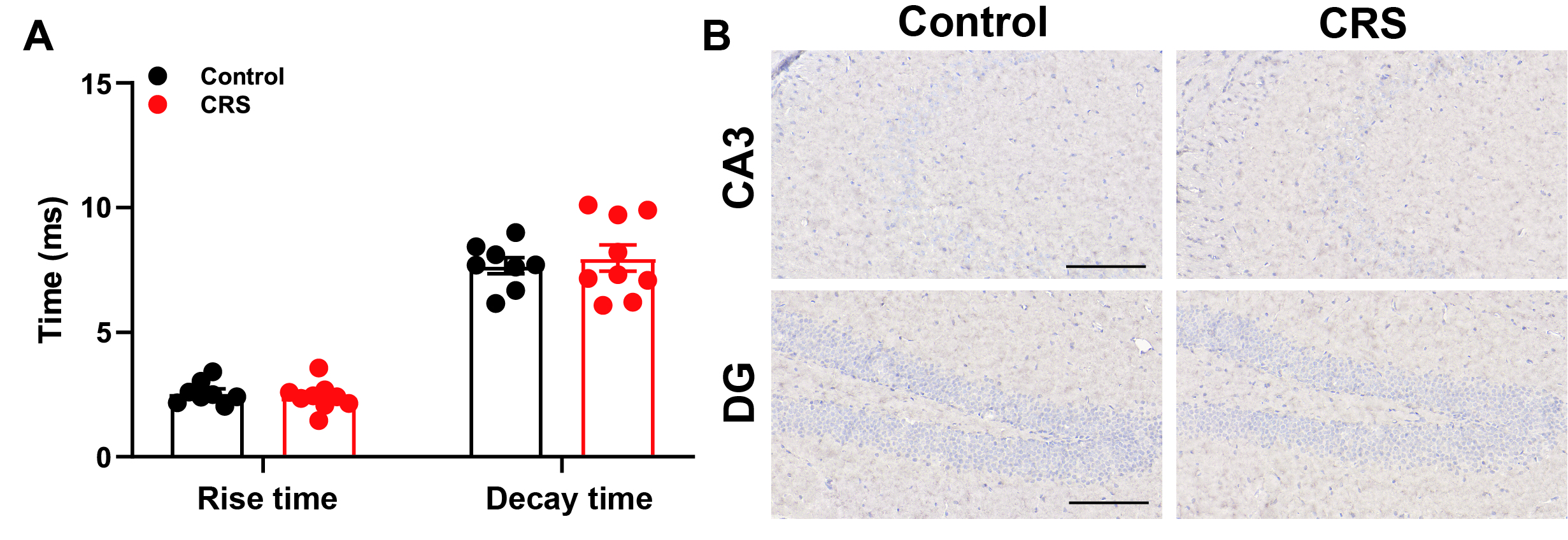
**

**Supplementary Fig. 1** (**A**) No effects of CRS on the mIPSCs rise time and decay time (n = 8-9 cells from 3-4 mice/group, Student’s test). (**B**) Immunohistochemistry against parvalbumin (PV)-positive GABAergic interneurons in the CA3 and DG. Scale bars: 200 μm. All data are shown as means ± SEM.

**
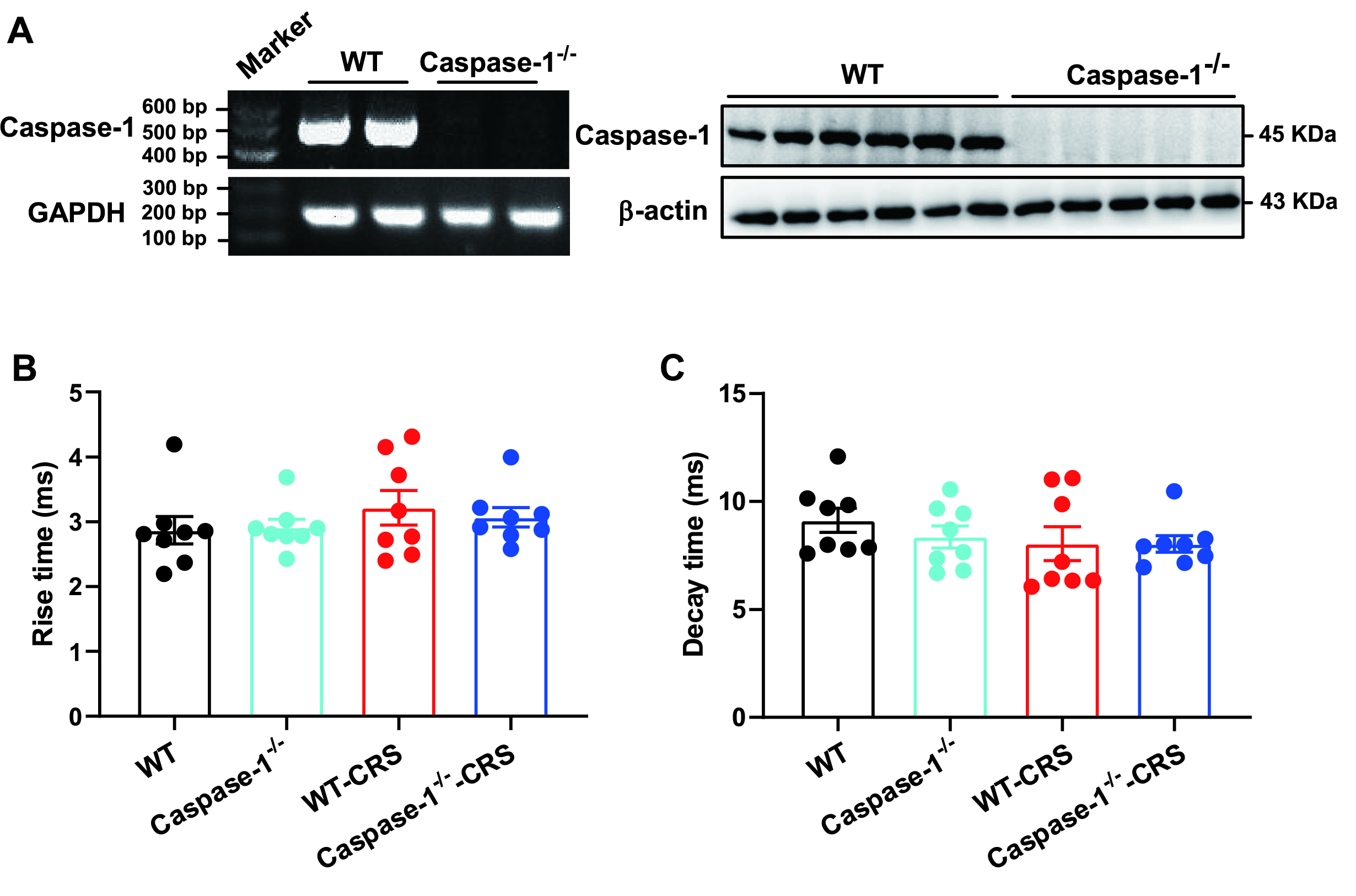
**

**Supplementary Fig. 2** (**A**) Representative reverse transcriptase-polymerase chain reaction (RT-PCR) and immunoblots from WT and Caspase-1^-/-^ mice. (**B and C**) Rise time (**B**, two-way ANOVA: CRS, F_(1,28)_ = 1.668, *p* > 0.05; Genotype, F_(1,28)_ = 0.067, *p* > 0.05; Interaction, F_(1,28)_ = 0.238, *p* > 0.05) and decay time (**C**, two-way ANOVA: CRS, F_(1,28)_ = 1.491, *p* > 0.05; Genotype, F_(1,28)_ = 0.450, *p* > 0.05; Interaction, F_(1,28)_ = 0.427, *p* > 0.05) of GABA_A_Rs-mediated mIPSCs from WT, WT-CRS, Caspase-1^-/-^ and Caspase-1^-/-^-CRS groups (n = 8 cells from 3-4 mice/group, two-way ANOVA, Bonferroni’s test). All data are shown as means ± SEM.


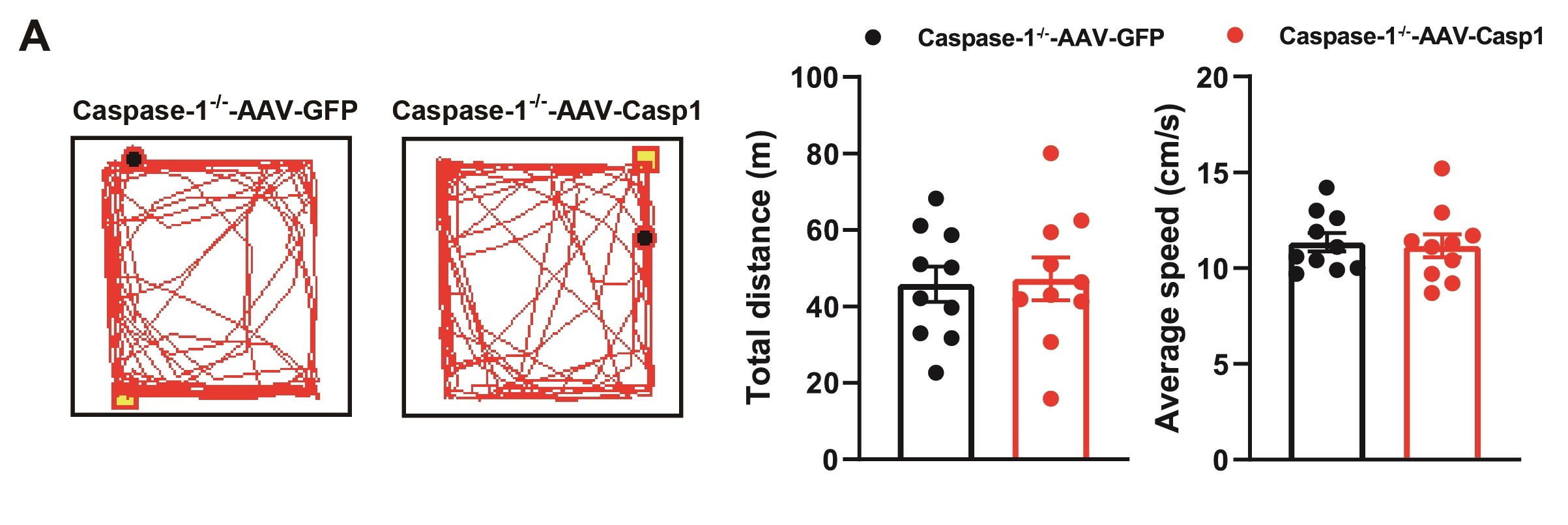


**Supplementary Fig. 3** There was no difference in the total distance and average speed from Caspase-1^-/-^-AAV-GFP and Caspase-1^-/-^-AAV-Casp1 groups in the OF test (n = 10 mice/group, Student’s test). All data are shown as means ± SEM.

**
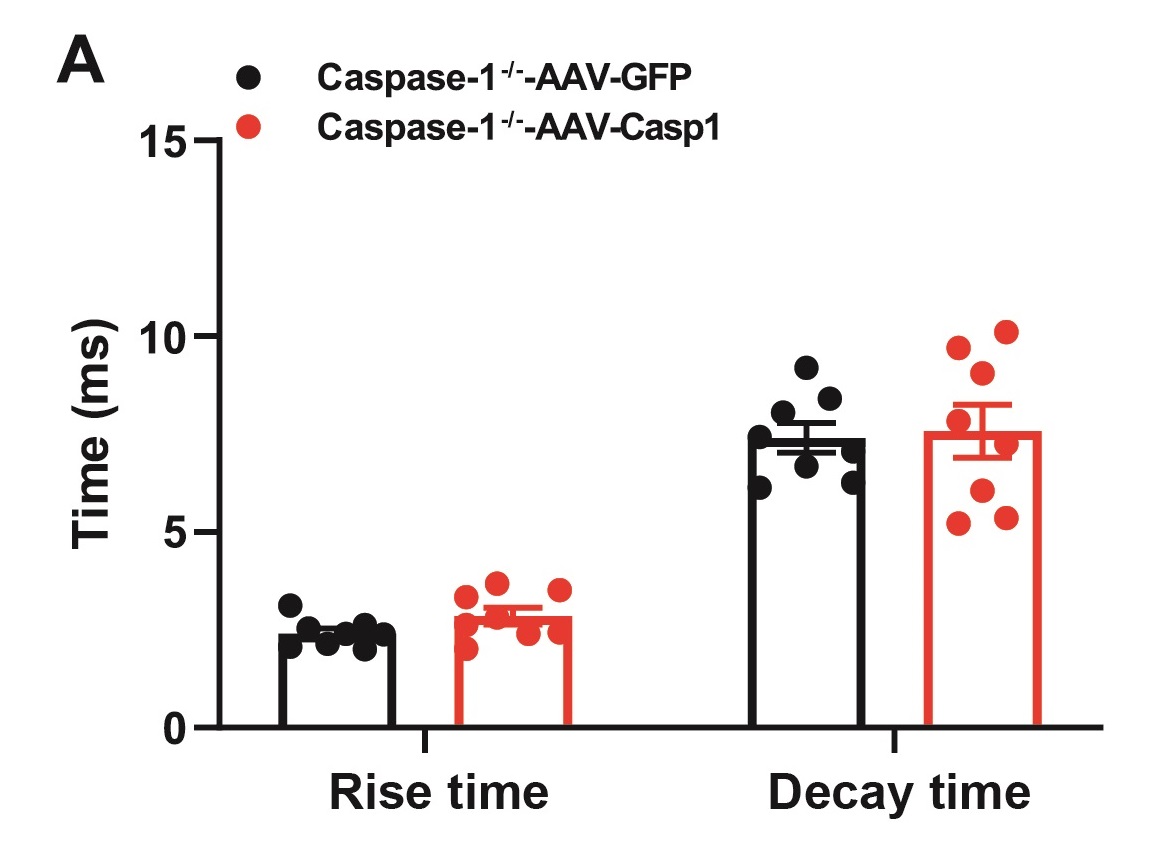
**

**Supplementary Fig. 4** There has no effect on the mIPSCs rise time and decay time from Caspase-1^-/-^-AAV-GFP and Caspase-1^-/-^-AAV-Casp1 groups (n = 8 cells from 3-4 mice/group, Student’s test). All data are shown as means ± SEM.


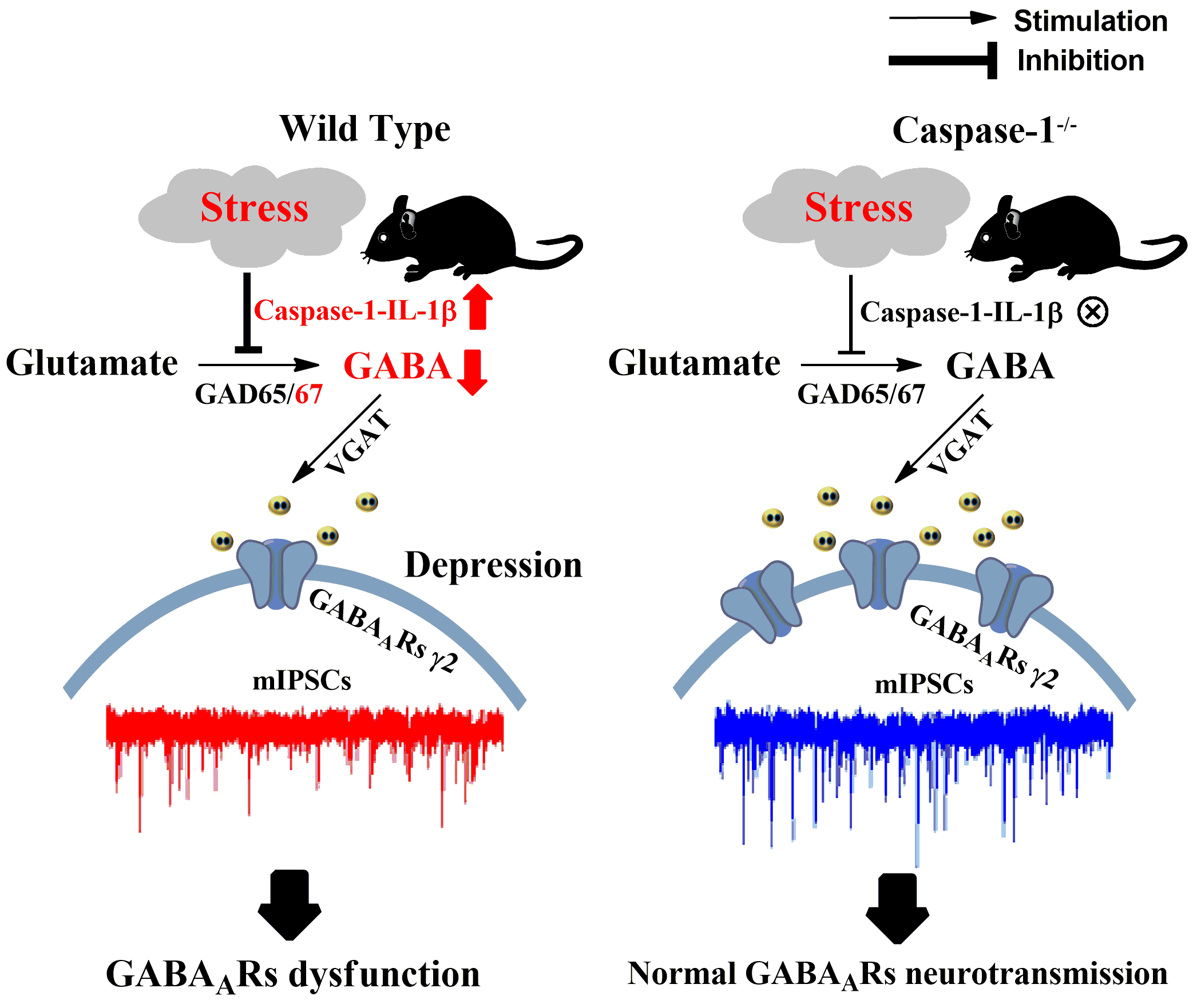


**Supplementary Fig. 5 Proposed model of caspase-1 in CRS-induced depression and GABAergic dysfunction.** CRS selectively increases the levels of caspase-1-IL-1β pathway in the hippocampus, subsequently decreases the levels of GAD67 and impairs GABA_A_Rs-mediated synaptic transmission, eventually leading to depressive-like behaviors in mice. Interestingly, gene deficiency of caspase-1 prevents CRS-induced depression-like behaviors and GABAergic dysregulation in the hippocampus, and reexpression of caspase-1 in the hippocampus of Caspase-1^-/-^ mice increases susceptibility to stress-induced depression and GABAergic dysfunction.

**Supplementary Tables**

**Supplementary Table 1. qPCR primers**

| Gene | Forward primer (5’ to 3’) | Reverse primer (5’ to 3’) |
| --- | --- | --- |
| Caspase-1  GAD65  GAD67  VGAT  GAPDH | CTTGGAGACATCCTGTCAGGG AGCCTCAACACACAAATGTCTGCTTCT  GGGTTCCAGATAGCCCTGAGCGA  TGCCATTCAGGGCATGTTCGT  ATGGTGAAGGTCGGTGTG | AGTCACAAGACCAGGCATATTCT TGGTCCCATACTCCATCATTCTGGCT  TGGCCTTGTCCCCTTGAGGCT  GGGGAATCGAGGAGCGCAGC  CATTCTCGGCCTTGACTG |

**Supplementary Table 2. Antibodies used in this study.**

| **Antibody** | **Company** | **Catalog No.** | **Dilution** | | **Application** | |
| --- | --- | --- | --- | --- | --- | --- |
| Anti-GAD67  Anti-Caspase-1  Anti-GABA_A_Rs γ2  Anti-Parvalbumin  Anti-β-actin  Anti-GAPDH  Anti-Parvalbumin | Merk Millipore  Merk Millipore  Bioss  Bioss  Absin  Absin  Bioss | MAB5406  06-503-I bs-4112R  bs-1299R  abs137975  abs132004  bs-1299R | | 1:500  1:1000  1:400  1:200  1:3000  1:3000  1:50 | | WB  WB  WB  WB  WB  WB  IH |

WB: Western blotting; IH, Immunohistochemistry.

**Supplementary Table 3. Statistical data for the results in each figure**
